# The impact of invasive plant management on the foraging ecology of the Warbler Finch (*Certhidea olivacea*) and the Small Tree Finch (*Camarhynchus parvulus*) on Galápagos

**DOI:** 10.1007/s10336-017-1481-4

**Published:** 2017-08-08

**Authors:** Nikolaus Filek, Arno Cimadom, Christian H. Schulze, Heinke Jäger, Sabine Tebbich

**Affiliations:** 1 Department of Behavioural Biology, University of Vienna, Althanstrasse 14, 1090 Vienna, Austria; 2 Department of Botany and Biodiversity Research, University of Vienna, Rennweg 14, 1030 Vienna, Austria; 3 Charles Darwin Foundation, Puerto Ayora, Santa Cruz, Galápagos Ecuador

**Keywords:** Darwin’s Finches, *Rubus niveus*, Invasive species, Habitat management, Foraging ecology, Restoration ecology

## Abstract

In recent decades, arboreal Darwin’s Finches have suffered from a dramatic population decline, which has been attributed to parasitism by the invasive botfly *Philornis downsi*. However, changes to their primary habitat caused by invasive plant species may have additionally contributed to the observed population decline. The humid cloud forest on Santa Cruz Island is a stronghold of arboreal Darwin’s Finches but has been invaded by blackberry (*Rubus niveus*). In some areas, manual control and herbicide application are used to combat this invasion, both causing a temporary removal of the entire understory. We hypothesized that the removal of the understory reduces the availability of arthropods, which are a main food source during chick rearing. We compared the foraging behaviour of Warbler Finches (*Certhidea olivacea*) and Small Tree Finches (*Camarhynchus parvulus*) at three study sites that varied in the degree of *R. niveus* invasion and the length of time since the last herbicide application. We used prey attack rate and foraging success as an index for food availability and predicted a lower attack rate and foraging success in areas that had recently been sprayed with herbicides. We found that both the invasion and the management of *R. niveus* influenced microhabitat use, foraging substrate and prey choice in both species. Contrary to our hypothesis, we did not find a lower attack rate or foraging success in the area with recent herbicide application. This may be explained by the finding that both species mainly foraged in the canopy but also used dead plant structures of the understory of the recently controlled area that resulted from the invasive plant management.

**Electronic supplementary material:**

The online version of this article (doi:10.1007/s10336-017-1481-4) contains supplementary material, which is available to authorized users.

## Zusammenfassung

**Der Einfluss von Managementmaßnahmen zur Kontrolle invasiver Pflanzen auf die Ernährungsökologie von Waldsängerfinken (** ***Certhidea olivacea*** **) und Kleinen Baumfinken (** ***Camarhynchus parvulus*** **) auf Galápagos**

Einige der arboreal lebenden Darwinfinken zeigten in den letzten Jahrzehnten einen dramatischen Populationsrückgang, verursacht durch die parasitische Fliege *Philornis downsi*. Ein weiterer Grund dafür könnte der Verlust von primärem Lebensraum aufgrund des Auftretens invasiver Arten und Managementmaßnahmen zur Kontrolle dieser sein. In den letzten verbliebenen Resten der feuchten *Scalesia*-Wälder auf Santa Cruz, Galápagos, welche durch die Invasion der Brombeerart *Rubus niveus* stark bedroht sind, geraten verschiedene Singvogelarten zusehends unter Druck. Der Einsatz von Herbiziden sowie das manuelle Entfernen von Pflanzen zur Kontrolle der Neophyten führen zu einer großflächigen Zerstörung des Unterwuchses. Eine frühere Studie zeigte einen reduzierten Bruterfolg der Waldsängerfinken (*Certhidea olivacea*) in Gebieten, in denen Managementmaßnahmen durchgeführt wurden. Daraus ergab sich die Annahme, dass der verringerte Bruterfolg durch eine Reduzierung des Nahrungsangebots verursacht wurde, die auf die Invasion von *R. niveus* und die Zerstörung des Unterwuchses zurückzuführen sei. Daher untersuchten wir das Furagierverhalten und den Jagderfolg des insektivoren Waldsängerfinken und des omnivoren Kleinen Baumfinken (*Camarhynchus parvulus*) an drei Standorten, die sich hinsichtlich Invasionsgrad von *R. niveus* und dem Zeitpunkt des Herbizideinsatzes unterschieden. Wir analysierten Beuteattacken und Nahrungserfolg als Indikatoren für Nahrungsverfügbarkeit und prognostizierten eine geringere Anzahl an Beuteattacken und geringeren Nahrungserfolg in erst kürzlich kontrollierten Gebieten. *R. niveus* und die Managementmaßnahmen beeinflussten das Furagierverhalten beider Arten in Bezug auf Mikrohabitatwahl, Substratnutzung und genutzter Nahrung. Entgegen unserer Vorhersage gab es keinen Effekt eines rezenten Herbizideinsatzes auf Beuteattacken und Nahrungserfolg der Finken. Dies könnte darauf zurückzuführen sein, dass die Vögel hauptsächlich in der Baumkrone furagiert haben und in rezent kontrollierten Gebieten nach wie vor den überwiegend aus totem Pflanzenmaterial bestehenden Unterwuchs nutzen.

## Introduction

The Galápagos Islands are one of the last oceanic archipelagos that still retain most of their original biodiversity. The Galápagos bird community has remained remarkably unaltered and only one of the 30 resident species has gone extinct since human settlement (Dvorak et al. 2004; Grant and Grant 2008; O’Connor et al. 2009; Carmi et al. 2016). However, the loss of primary habitat due to agriculture and the spread of invasive species has led to increasing pressures, affecting several land bird species, especially Darwin’s Finches.

Bird counts between 1997 and 2010 on Santa Cruz Island, the island with the highest population of humans in Galápagos, revealed that six out of nine investigated passerines had declined significantly (Dvorak et al. 2011). The most dramatic decline was observed in the insectivorous Warbler Finch (*Certhidea olivacea*), which is the smallest of the Darwin’s Finches. From 1997 to 2010, its population on Santa Cruz Island decreased by 45% in the humid native forest (*Scalesia* forest) and by 85% in the agricultural zone. The closely related sympatric Small Tree Finch (*Camarhynchus parvulus*) has shown a population decline since 2005 (Cimadom et al. 2014). We investigated the breeding success of both species in the *Scalesia* forest, which is the area with the highest density of arboreal Darwin’s Finches, and found that both species suffered from high brood loss due to the invasive parasitic fly *Philornis downsi* (Cimadom et al. 2014). The larval stage of this obligate bird parasite develops in birds’ nests and sucks blood from the nestlings (Dudaniec and Kleindorfer 2006; Fessl et al. 2006; O’Connor et al. 2010a, b). Parasitism by *P. downsi* has probably caused population declines of several Darwin’s Finch species (Fessl et al. 2010; O’Connor et al. 2010a, b; Young et al. 2013; Rodríguez and Fessl 2016).

However, our data also point towards a negative influence of the habitat change (Cimadom et al. 2014). On Santa Cruz Island, the *Scalesia* forest had been reduced to only 1% of its original distribution by previous agricultural activity (Mauchamp and Atkinson 2009), and more recently has been invaded by different introduced plant species (Renteria et al. 2012). Of these, *Rubus niveus* (blackberry) has had the strongest negative effect on the original plant species composition. After its introduction on Santa Cruz Island in 1986, it spread rapidly and became very abundant in the last remnants of the *Scalesia* forest at ‘Los Gemelos’ approximately 10 years ago (Rentería and Buddenhagen 2006; Renteria et al. 2012). Uninvaded *Scalesia* forest with natural understory has disappeared entirely (personal observation Cimadom and Tebbich). In some areas of the *Scalesia* zone, the Galápagos National Park Directorate (GNPD) manually controls *R. niveus* with machetes and subsequently applies herbicides onto the regrowth to assist in the natural regeneration of the forest. The manual control caused a temporary removal of the understory, and we found that the breeding success of Warbler Finches was significantly lower in areas in which *R. niveus* had been recently controlled (Cimadom et al. 2014). Since arthropods use plants for refuge and/or for food, the removal of the understory may reduce their abundance (Boada 2008). Studies conducted in British and Canadian agricultural systems showed that the use of herbicides can cause declines in plant diversity that lead to a reduction in arthropods (Moreby and Southway 1999; Morris et al. 2005; Boutin et al. 2012). Like most small passerines, Darwin’s Finches depend on insects for chick rearing (Betts 1955; Stutchbury and Morton 2001). Therefore a lower insect abundance or availability might contribute to the lowered breeding success of the Warbler Finch in blackberry control areas (Cimadom et al. 2014). We hypothesized that habitat changes, due to both invasion by *R. niveus* and the control measures used, reduce the availability of arthropods and change their micro-spatial distribution (e.g. across forest strata) and thus influence the foraging behaviour of the two study species.

Sampling arthropod abundance may not be the most accurate way to assess food availability for insectivorous birds because arthropods differ in mobility, crypsis and palatability (Sherry 1984; Holmes and Schultz 1988; Hutto 1990). In combination, these factors will influence the prey availability that birds are actually experiencing. Thus, it may be more effective to measure bird behaviours that reflect prey availability (Hutto 1990; Lovette and Holmes 1995). Prey attack rate has been suggested and used as appropriated measure by several authors (Hutto 1990; Lovette and Holmes 1995; Olsson et al. 2001; Oyugi et al. 2012). However, in extractive foraging, birds are searching for hidden food, which makes it difficult to distinguish between search manoeuvres and prey attacks. Therefore, prey attack rates do not necessarily reflect prey availability. Foraging success (number of food items consumed per time) could be a more accurate measure for food availability. However, measuring foraging success is often not feasible, if birds are observed from a distance. The Small Tree Finch and the Warbler Finch are partly extractive foragers and can be observed from a very close distance. Therefore, we used both, prey attack rate, measured as foraging techniques performed per foraging time, and foraging success, measured as foraging items ingested per foraging time as an index for food availability and abundance.

In the present study, we compared microhabitat use, foraging substrates, prey choice, prey attack rate and foraging success of Small Tree Finches and Warbler Finches in three different habitat conditions that varied in the degree of *R. niveus* invasion and the length of time since the last herbicide application. We hypothesized that food availability would be lower in areas where herbicides had been recently applied, since manual and chemical control of *R. niveus* altered the vegetation structure and reduced plant biomass. Thus, we predicted a lower prey attack rate and foraging success in the recently controlled area. Furthermore, we assumed that prey attack rate and foraging success in different microhabitats (e.g. near ground, understory, canopy) would differ between invaded and managed sites and this difference would be most pronounced in the understory of the recently controlled area.

## Methods

### Study area

**Fig. 1:**
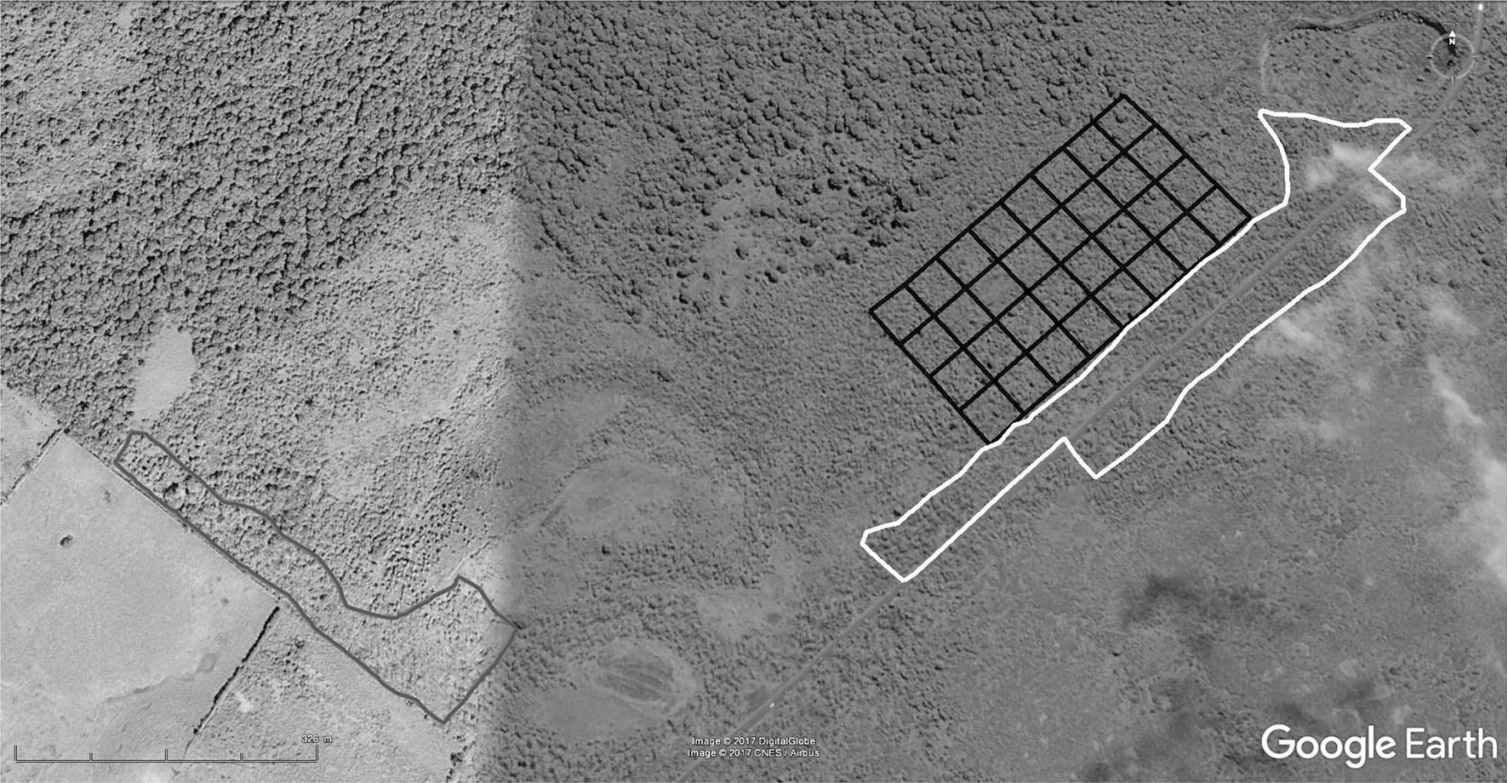
Map (Google Earth™) showing part of the *Scalesia* zone with the main road at Los Gemelos, Santa Cruz (0°37′34″S, 90°23′10″W). The different study sites are framed as followed: in *black* ‘invaded’ area (8 ha), in *grey* ‘recently controlled’ area (3.2 ha) and in *white* area with long-term management (6.7 ha)

The study was conducted from January–April 2014 in the *Scalesia* forest near ‘Los Gemelos’ in the humid highlands of Santa Cruz Island (0°37′34″ S, 90°23′10″ W; 500–600 m a.s.l., Fig. 1). The forest is dominated by the endemic tree species *Scalesia pedunculata* (Asteraceae) but has been invaded by several introduced plant species, predominantly by *R. niveus* Thunb. (blackberry, Rosaceae), *Tradescantia fluminensis* Vell. (wandering jew, Commelinaceae), *Cestrum auriculatum* L’Hér. (sauco, Solanaceae) and *Piper peltatum* L. (Piperaceae).

We conducted foraging observations in three study areas with different *R. niveus* management regimes (=habitat conditions): (1) an area heavily invaded by *R. niveus* and other introduced plant species, which had not been subjected to any control measures (referred to as ‘invaded’, 8 ha), (2) an area in which invasive plant species had been manually removed and subsequently treated with herbicides six months prior to data collection (referred to as ‘recently controlled’, 3.2 ha) and (3) an area in which the initial manual and chemical control of *R. niveus* (and other invasive plant species) had been followed up by localized herbicide applications on the invasive species’ regrowth since 2010 (referred to as ‘long-term management’, 6.7 ha). The long-term management measures in this area resulted in an open understory.

The manual and chemical control of *R. niveus* consisted of the GNPD cutting down the plants with machetes and a subsequent herbicide application (mixture of glyphosate and COMBO© = picloram and metsulfuron-methyl) on the re-grown shoots. These control measures led to the removal of almost the entire understory, with dead *R. niveus* branches and other dead plant structures left in the ‘recently controlled’ areas.

### First foraging observations

We monitored the foraging behaviour of the more generalist Small Tree Finch and the insectivorous Warbler Finch during their breeding season from January–April 2014 in the three habitat conditions, following the classification system of Remsen and Robinson (1990) and Tebbich et al. (2004). To access the study area, a 50 × 50 m grid trail system was cut into the dense invaded understory vegetation by GNPD rangers. Observations were carried out from 06:30 to 12:30 a.m. The first time that an observed bird was seen foraging, is referred to as ‘first foraging observation’. We used first foraging observations to collect data on foraging substrate, foraging technique, foraging height, microhabitat (ground, understory up to 3.5 m and canopy above 3.5 m) and prey type (Small Tree Finch: *n* = 248; Warbler Finch: *n* = 364). To minimize the possibility of repeated observations of the same bird individual during a day, we used trails to explore different areas in the three habitat conditions. However, except in the case of banded birds, it was not possible to exclude the possibility of individuals entering the data set more than once.

Foraging substrates were categorized as ‘dead leaf’ (still attached to branch), ‘leaf’, ‘moss’, ‘twig’, ‘bark’, ‘*R. niveus*’, ‘*C. auriculatum*’, ‘*Scalesia* seed’ (seed stems of the *Scalesia* tree), ‘herb’, ‘soil’ (e.g. seeds on the ground) and ‘others’, following Tebbich et al. (2004).

Foraging techniques were categorized as ‘probe’ (rapidly inserting the beak into moss or dead leaves), ‘bite’ (chipping off parts of bark or removing moss with the beak), ‘glean’ (taking food directly from a substrate surface), ‘sally’ (aerial attack) and ‘feed’ (only used on plant food sources like ‘nectar’, ‘fruit’ and ‘seed’), following Tebbich et al. (2004).

Where possible, animal prey types were identified to the order-level (‘Coleoptera’, ‘Hemiptera’, ‘Orthoptera’ and ‘Lepidoptera’). Non-identified arthropods were categorized as ‘other arthropods’. In ‘Lepidoptera’, we distinguished between ‘caterpillars’ and ‘moths’, since caterpillars are an important food source during chick rearing in most passerine bird species (Thiollay 1988; Greenberg 1995). Plant food types, pollen and nectar, both food sources of flower visiting birds, were combined into the category ‘nectar’. Other categories were ‘fruit’ and ‘seed’.

### Prey attack rate and foraging success

To measure the prey attack rate and foraging success, we followed individual birds and recorded all foraging events on a tape recorder with an integrated stopwatch (Olympus Digital Voice Recorder VN-712PC) during a continuous focal observation (Small Tree Finch: *n* = 150; Warbler Finch: *n* = 191). Following Lovette and Holmes (1997) we defined prey attack rate as the number of performed foraging techniques per minute foraging time and foraging success as the number of food items (arthropods and plants) ingested per minute foraging time. Observations began with the first foraging event of the focal individual and ended when the bird switched microhabitat or flew out of sight. Since Darwin’s Finches sometimes combine foraging with short singing and preening bouts, observations did not end when birds briefly engaged in other behaviours. However, when birds clearly stopped foraging and engaged in other behavioural activities that lasted longer than several seconds observation stopped. Thus, foraging time corresponds to length of observation in which birds predominantly foraged. Observed foraging bouts were between 13 to 703 s long (median 157 s). The vegetation density is much higher in heavily invaded habitat condition than in the recently controlled and in the long-term managed areas, which may affect observability. However, the duration of foraging bouts did not differ significantly among the three habitat conditions (Kruskal–Wallis test; Small Tree Finch: *H* = 1.57, *df* = 2, *p* = 0.455; Warbler Finch: *H* = 3.37, *df* = 2, *p* = 0.186, Online Resource Table S1). Additionally, the duration of foraging bouts did not differ significantly between the two microhabitats analysed (Mann–Whitney *U* test, Small Tree Finch: *U* = 2016.5, *p* = 0.161; Warbler Finch: *U* = 4410.5, *p* = 0.211, Online Resource Table S2).

### Stomach contents

Since nestling diets may differ from adult diets and small prey items can often not be identified in foraging observations but in stomach contents, we analyzed stomachs of dead chicks. These were collected from unsuccessful nests of both Darwin’s Finch species. Dead chicks were transferred to the lab and individually stored in 70% alcohol (Small Tree Finch: *n* = 26; Warbler Finch: *n* = 16). The stomach was excised, cut open, the content placed into a petri dish and then dried for at least 10 min and identified using a stereomicroscope. Stomach contents were categorized as ‘stones’, ‘seeds’, ‘invertebrate eggs’, ‘ants’, ‘beetles’, ‘caterpillars’, ‘moths’, ‘cicadas’ and ‘spiders’. ‘Stones’, ‘seeds’ and ‘invertebrate eggs’ were counted. To estimate the number of insects, we used the number of heads, pairs of mandibles or other bigger chitin structures. Since parts of insects may be missing, the results for arthropods are descriptive only. Unidentified material was not quantified and was excluded from the results.

### Statistical analysis

We used chi-square tests to test for differences in foraging technique, substrate, microhabitat use and prey types among the three different habitat conditions. When tests indicated significant effects, we subsequently calculated pair-wise comparisons between habitat conditions and applied a Bonferroni correction to account for multiple testing. Since both Darwin’s Finch species rarely used the ground, we excluded this microhabitat from the statistical analysis. In the Warbler Finch we rarely observed the use of plant food and thus we excluded plant food sources from the statistical analysis for this species. If cell frequencies were below 10, Yates’ continuity correction was calculated. All tests were two-tailed with an alpha level of 0.05. We calculated generalized linear models (GLMs; with normal error distribution and log link function) to evaluate the effects of habitat conditions, microhabitat and the interaction term habitat conditions × microhabitat on the prey attack rate and foraging success, respectively, of both Small Tree Finch and Warbler Finch using Statistica version 7.1 software (StatSoft, Inc. 1984–2005). For the Small Tree Finch, we calculated models using values for prey attack rate and foraging success based on all consumed food and considering only animal food. GLMs evaluating effects on prey attack rate and foraging success of the Warbler Finch only considered animal food.

## Results

### Foraging technique and substrate

Small Tree Finches (*n* = 246) most frequently used the techniques ‘probe’ (50%) and ‘feed’ (36%) and to a much lower percentage ‘glean’ (10%) and ‘bite’ (4%). Warbler Finches (*n* = 363) were observed mainly using the techniques ‘probe’ (50%) and ‘glean’ (43%). Other techniques used were ‘sally’ (4%), ‘feed’ (2%) and ‘bite’ (1%).

‘Dead leaf’ was the substrate used most frequently by both species (Small Tree Finch: 43%; Warbler Finch: 36%, Table 1), and they almost exclusively foraged on dead leaves of the endemic *S. pedunculata* tree (Small Tree Finch: 96%; Warbler Finch: 85%). Small Tree Finches additionally used ‘*Scalesia* seed’, ‘herb’, *‘C. auriculatum*’ and ‘soil’. Other important substrates for Warbler Finches were ‘leaf’, ‘moss’, ‘twig’, ‘bark’ and ‘*R. niveus*’ (Table 1).

**Table 1:** Different substrates used (percentages) by the generalist Small Tree Finch (*C. parvulus*) and the insectivorous Warbler Finch (*C. olivacea*) over all three different habitat conditions

Species	Frequency of substrate use (%)											
	Dead leaf	Leaf	Moss	Twig	Bark	*R. niveus*	Others	*Scalesia* seed	Herb	*C. auriculatum*	Soil	*n*
Small Tree Finch	43	8	9	3	0	8	2	15	5	4	3	246
Warbler Finch	36	26	17	13	5	3	1	0	0	0	0	363

Sample size is the number of foraging observations

### Microhabitat use

Canopy was the most frequently used microhabitat for both species, followed by understory and ground. 69% of all first foraging observations of Small Tree Finches were made in the canopy, 28% in the understory and 3% on the ground. For Warbler Finches, 63% of first foraging observations were observed in the canopy, 37% in the understory and none on the ground.

The microhabitat use of Small Tree Finches did not differ among the three habitat conditions (χ^2^-test, *n* = 239, *df* = 2, χ^2^ = 2.65, *p* = 0.266, Table 2). For the Warbler Finch, we found a significant difference in microhabitat use among the habitat conditions (χ^2^-test, *n* = 360, *df* = 2, χ^2^ = 6.21, *p* = 0.045, Table 2). The post hoc analysis revealed that Warbler Finches used the understory in the ‘invaded’ area more often than in the ‘long-term management’ area (χ^2^-test, *n* = 298, *df* = 1, χ^2^ = 5.94, *p* = 0.048, all other comparisons were non-significant).

**Table 2:** Differences in microhabitats used by the Small Tree Finch (*C. parvulus, n* = 239) and the Warbler Finch (*C. olivacea, n* = 360) among the three habitat conditions (‘invaded’, ‘recently controlled’, ‘long-term management’)

Species Habitat condition	Frequency of microhabitat use (%)		
	Understory	Canopy	*n*
Small Tree Finch			
Invaded	30	70	87
Recently controlled	39	61	36
Long-term management	25	75	116
Warbler Finch			
Invaded	43	57	150
Recently controlled	40	60	62
Long-term management	30	70	148

Sample size is the number of foraging observations

### Diet and prey items

Prey could be identified in 326 out of 612 first foraging observations. For Small Tree Finches, animals were the food source in 34% of the observations (*n* = 133) and for Warbler Finches in 96% (*n* = 193). Main animal prey types for Small Tree Finches and Warbler finches were ‘other arthropods’, ‘caterpillars’ and ‘moth’ (Table 3). Plant food resources of Small Tree Finches were more diverse than of Warbler Finches. They used ‘nectar’ of *R. niveus* (73%), *C. auriculatum* (18%) and *Passiflora sp.* (9%), ‘fruit’ of *R. niveus* (58%) and *C. auriculatum* (42%). Small Tree Finches also collected the ‘seed’ of seed stems of *S. pedunculata* (64%), as well as the seeds of various herb species (24%) and seeds from the ground (12%). The only plant food source of the Warbler Finch was ‘nectar’ of *R. niveus*.

**Table 3:** Relative frequency of different prey types foraged by the Small Tree Finch (*C. parvulus*) and the Warbler Finch (*C. olivacea*) over all three different habitat conditions

Species	Frequency of foraged prey types (%)						
	Other invertebrates	Caterpillar	Moth	Nectar	Fruit	Seed	*n*
Small Tree Finch	21	10	3	8	14	44	133
Warbler Finch	59	30	7	4	0	0	193

Sample size is the number of foraging observations

We did not find a significant difference in the proportion of plant to animal food among habitat conditions for Small Tree Finches (χ^2^-test, *n* = 126, *df* = 2, χ^2^ = 5.58, *p* = 0.061, Table 4). However, the proportion of utilized plant food types did not differ significantly among habitat conditions (χ^2^-test corrected for low expected frequencies: *n* = 81, *df* = 4, Yates’ χ^2^ = 17.28, *p* = 0.002, Table 4). The post hoc analysis revealed that in the ‘invaded’ area, Small Tree Finches foraged ‘nectar’ and ‘fruit’ more often than in the ‘long-term management’ area (χ^2^-test: *n* = 70, *df* = 2, χ^2^ = 16.81, *p* < 0.001, all other comparisons were non-significant). In an analysis of types of animal food, we did not find a significant difference among habitat conditions (χ^2^-test corrected for low expected frequencies: *n* = 45, *df* = 4, Yates’ χ^2^ = 4.93, *p* = 0.294, Table 4).

**Table 4:** Relative frequency of animal and plant prey in general and different animal prey and plant prey types only foraged by the omnivorous Small Tree Finch (*C. parvulus*) and the insectivorous Warbler Finch (*C. olivacea*) in the three different habitat conditions (‘invaded’, ‘recently controlled’ and ‘long-term management’

Species Habitat condition	Frequency of used prey (%)								
	Animal in general	Plant in general	Other arthropods	Caterpillar	Moth	Nectar	Fruit	Seed	*n*
Small Tree Finch									
Invaded	23	77	75	17	8	27	32	41	53
Recently controlled	40	60	75	0	25	0	8	92	20
Long-term management	42	58	52	44	4	0	14	86	60
Warbler Finch									
Invaded	90	10	58	27	5	100	0	0	81
Recently controlled	100	0	54	38	8	0	0	0	26
Long-term management	100	0	62	30	8	0	0	0	86

Sample size is the number of foraging observations

An analysis of the animal food types consumed by Warbler Finches showed no significant difference among the three habitat conditions (χ^2^-test, *n* = 183, *df* = 4, χ^2^ = 1.25, *p* = 0.870, Table 4).

### Prey attack rate and foraging success

In both species prey attack rate was neither affected by habitat conditions nor by microhabitat or the interaction term habitat condition × microhabitat (Table 5). However, when only considering animal food, microhabitat proved to significantly affect prey attack rate of Small Tree Finches with a generally higher rate in the canopy layer (Table 5).

**Table 5:** Results of GLMs testing for effects of habitat condition, microhabitat and the interaction term habitat condition × microhabitat on prey attack rate of the Small Tree Finch (*C. parvulus*), including and excluding plant food and the Warbler Finch (*C. olivacea*), excluding plant food

Effect	*df*	MS	*F*	*p*
Small Tree Finch				
Including plant food				
Constant	1	141.55	400.69	<0.0001
Habitat condition	2	0.72	2.04	0.1341
Microhabitat	1	0.49	1.39	0.2411
Habitat condition × microhabitat	2	0.23	0.66	0.5166
Error	134	0.35		
Excluding plant food				
Constant	1	91.98	314.60	<0.0001
Habitat condition	2	0.88	1.50	0.2310
Microhabitat	1	2.23	7.62	0.0075
Habitat condition × microhabitat	2	0.29	1.01	0.3711
Error	64	0.29		
Warbler Finch				
Excluding plant food				
Constant	1	300.66	1665.13	<0.0001
Habitat condition	2	0.30	1.65	0.1957
Microhabitat	1	0.12	0.69	0.4070
Habitat condition × microhabitat	2	0.18	1.02	0.3628
Error	178	0.18		

**Fig. 2:**
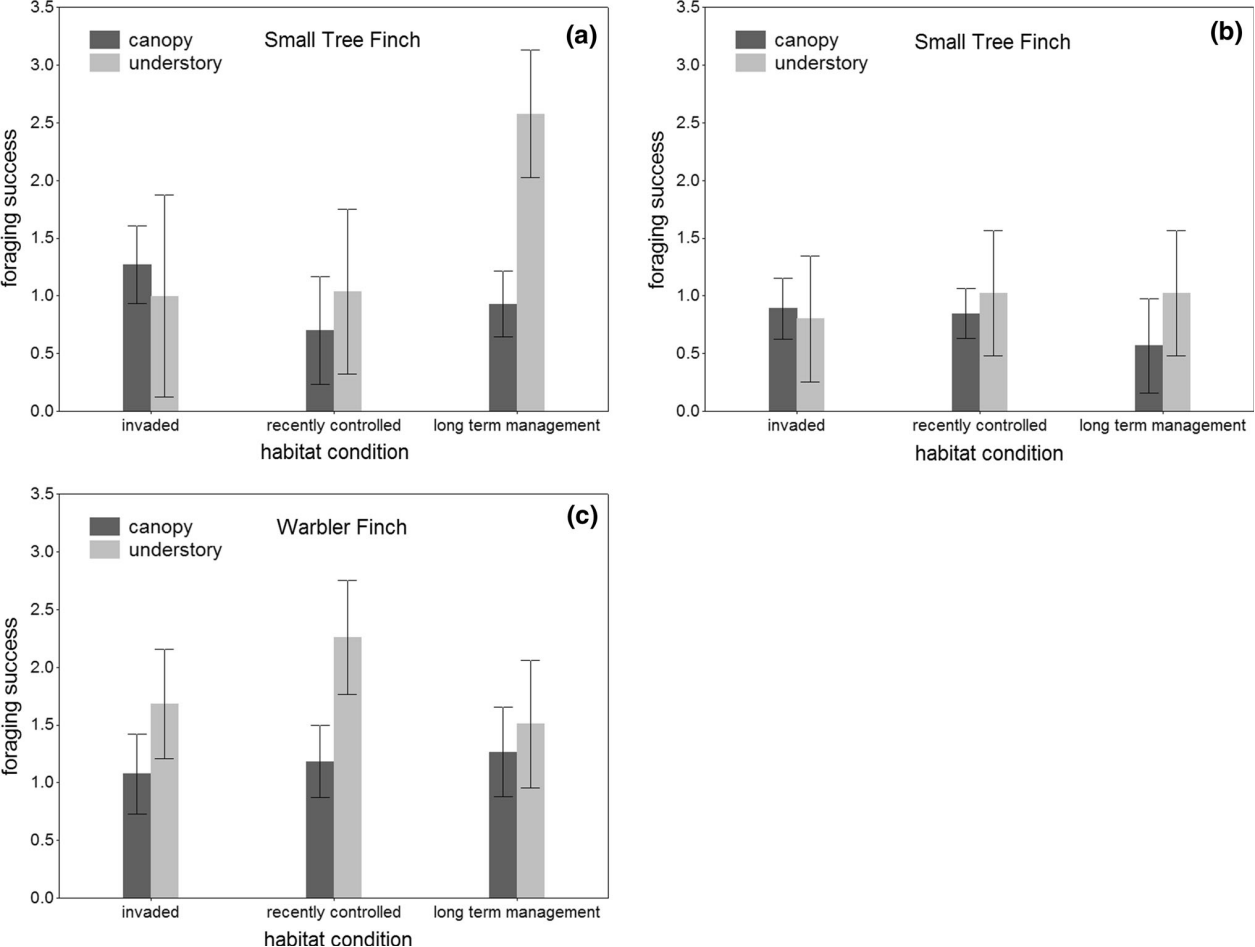
Least square means (±95% CI) of foraging success of the Small Tree Finch (*C. parvulus*), **a** including and **b** excluding plant food, and the Warbler Finch (*C. olivacea*), **c** excluding plant food, in canopy and understory in the three different habitat conditions

For the Small Tree Finch, habitat condition, microhabitat and the interaction term all proved to have a significant effect on its foraging success (Table 6). Foraging success in the understory of the ‘long-term management’ area was more than twice as high as in the canopy of the same area and also in the understory of the two other habitat conditions (Fig. 2a). Remarkably none of these effects remained significant, when only considering animal food (Fig. 2b). The foraging success of the Warbler Finch was significantly higher in the understory than in the canopy (Fig. 2c), but it was not affected by habitat conditions or the interaction term habitat condition × microhabitat (Table 6).

**Table 6:** Results of GLMs testing for effects of habitat condition, microhabitat and the interaction term habitat condition × microhabitat on foraging success of the Small Tree Finch (*C. parvulus*), including and excluding plant food and the Warbler Finch (*C. olivacea*), excluding plant food

Effect	*df*	MS	*F*	*p*
Small Tree Finch				
Including plant food				
Constant	1	40.92	295.23	<0.0001
Habitat condition	2	0.69	5.01	0.0079
Microhabitat	1	0.65	4.68	0.0322
Habitat condition × microhabitat	2	0.73184	5.28	0.0061
Error	143	0.13862		
Excluding plant food				
Constant	1	14.41	125.39	<0.0001
Habitat condition	2	0.06	0.53	0.5909
Microhabitat	1	0.12	1.07	0.3053
Habitat condition × microhabitat	2	0.09	0.81	0.4498
Error	71	0.11		
Warbler Finch				
Excluding plant food				
Constant	1	363.47	274.94	<0.0001
Habitat condition	2	2.20	1.66	0.1922
Microhabitat	1	16.65	12.59	0.0005
Habitat condition × microhabitat	2	2.30	1.74	0.1784
Error	185	1.32

### Stomach contents

**Fig. 3:**
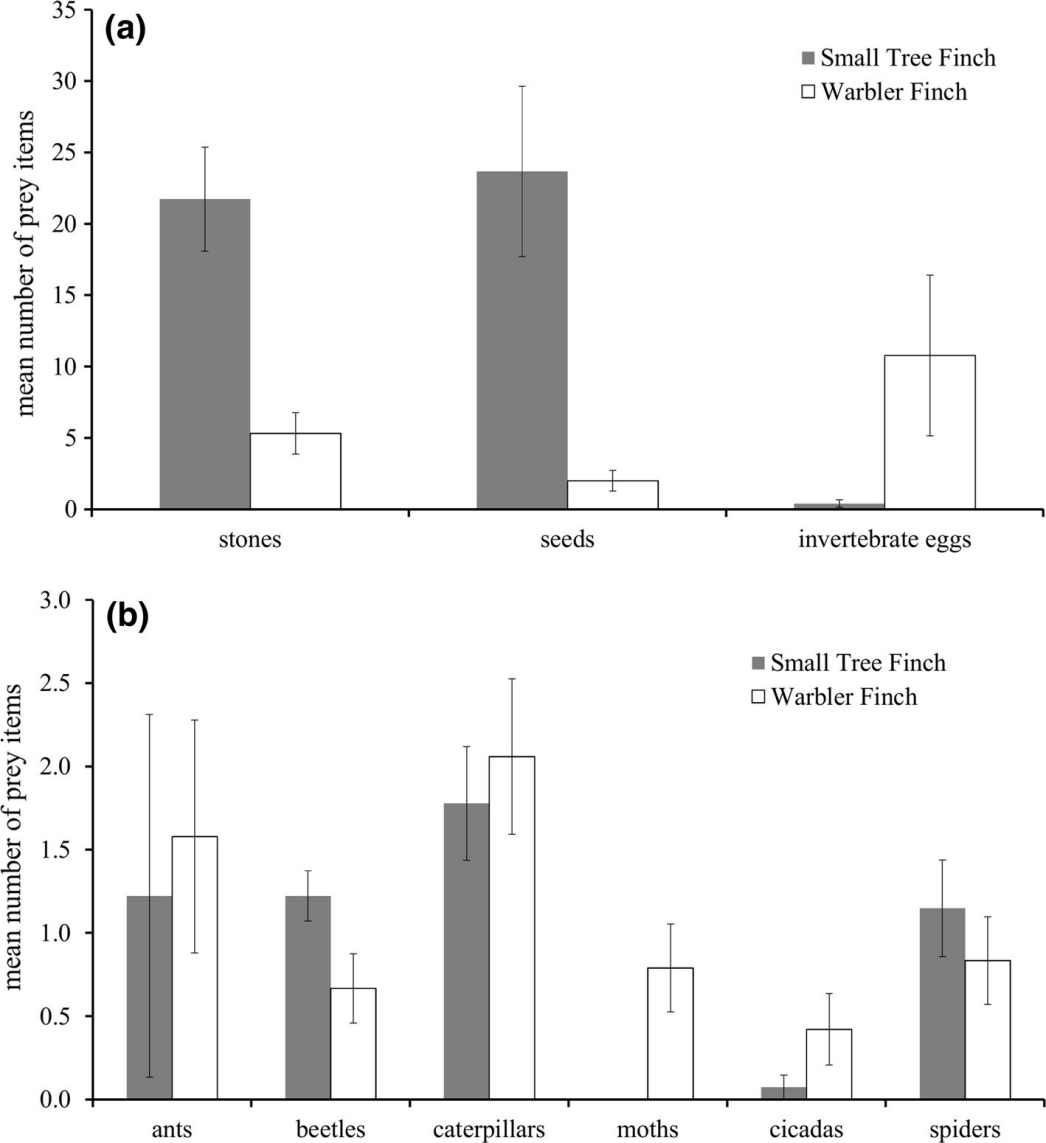
Mean number (±SE) of **a** invertebrate eggs, seeds and stones, and **b** animal prey items found in stomachs of dead chicks of the Small Tree Finch (*C. parvulus*) and the Warbler Finch (*C. olivacea*)

For both Darwin’s Finch species, ‘caterpillars’ was the most abundant arthropod category. Furthermore, we found a high number of ‘seeds’, ‘stones’ and beetles of the family Curculionidae in the stomachs of Small Tree Finch chicks, whilst Warbler Finch chicks had many ‘invertebrate eggs’ in their stomachs. Small Tree Finch chicks were additionally fed with ‘beetles’, ‘spiders’, ‘ants’ and ‘cicadas’ and Warbler Finch chicks with ‘ants’, ‘spiders’, ‘moths’, ‘beetles’ and ‘cicadas’. The mean number of ‘seeds’, ‘stones’ and ‘invertebrate eggs’, along with the mean number of the most common arthropod taxa found in the stomachs of dead chicks, are depicted in Fig. 3a, b (Small Tree Finch: *n* = 26, Warbler Finch: *n* = 19).

## Discussion

The foraging success of Small Tree Finches was twice as high in the understory of the ‘long-term management’ area than in the understory of the ‘recently controlled’ and ‘invaded’ areas. The foraging success of the Small Tree Finch in the understory of the ‘long-term management’ area was also twice as high as in the canopy of the same area. However, this effect disappeared when animal food was considered only. A possible explanation could be that across all three habitat conditions, the ‘long-term management’ area represented the most natural *Scalesia* forest with a diverse spectrum of native plant species. The understory of this area probably provided more plant food sources for Small Tree Finches.

Contrary to our hypothesis, we did not find a lower prey attack rate or lower foraging success in either bird species in the ‘recently controlled’ area compared to the other areas. One possible explanation for this result is that both species foraged mainly in the canopy, which is the microhabitat least affected by the control measures of the Galápagos National Park Directorate. The main foraging technique used by both species in the canopy was ‘probe’, applied to the substrates ‘dead leaf’ and ‘moss’. *Scalesia* trees provide several suitable microhabitats for a variety of insects, such as dead leaves remaining attached to branches, moss and bark. Attached dead leaves play an important role for several Neotropical insectivorous birds with foraging techniques adapted to utilise this particular feeding niche (Remsen and Parker 1984; Canaday 1996). However, the search for arthropods hidden in curled dead leaves comes at a high trial and error cost, resulting in a higher prey attack rate but not higher foraging success in the canopy. In line with this, the prey attack rate of Small Tree Finches was higher in the canopy than in the understory, but the foraging success was similar in both vegetation strata. Nonetheless, foraging in the canopy may be more profitable because attached dead leaves often contain large and soft prey items like caterpillars and orthopterans with a high energy and protein content, which is important during chick rearing (Remsen and Parker 1984; Rosenberg 1997; Sutherland et al. 2004). A qualitative search revealed that during daytime, many spiders, beetles, caterpillars and orthopterans hide in dead *Scalesia* leaves still attached to the branches.

Even when considering the understory alone, we did not find a difference in foraging success among the three habitat conditions for the Warbler Finch. Although the manual and chemical control of *R. niveus* in the ‘recently controlled’ area removed the understory, it left dead plant structures behind. We observed many birds using these dead plant structures as foraging substrates. The ‘recently controlled’ area offered many open patches with direct sun exposure for thermophilic arthropods. Additionally, flying insects like moths are known for their dispersal ability in search for new feeding grounds and/or reproduction sites (Young 1997; Dettner and Peters 2011), and hence are capable of rapidly recolonizing recently controlled areas.

Both the invasion of *R. niveus* and the management of invasive plants influenced microhabitat use, foraging substrate and prey choice of the two Darwin’s Finch species in this study. *R. niveus* and other introduced plant species were frequently used as substrates and food sources in the ‘invaded’ area. In this area, Warbler Finches used the microhabitat understory significantly more often than in the ‘long-term management’ area. A reason for this may be that in the ‘long-term management’ area, the understory had been reduced to a few native shrubs and herbaceous species. Additionally, GNPD rangers had removed dead plant structures from this area and structures which were used by Warbler Finches as foraging substrate in the ‘recently controlled’ area. The microhabitat use of Small Tree Finches did not differ among the three habitat conditions, probably because overall they were more frequent users of the canopy.

The invasion of *R. niveus* influenced the diet of both studied species. In the ‘invaded’ area, the composition of plants used as food differed significantly from the other two habitat conditions, whilst arthropod prey types were similar in all habitat conditions. In our study, Small Tree Finches foraged ‘nectar’ and ‘fruit’ more often in the ‘invaded’ area than in the other habitat conditions. In ‘recently controlled’ and ‘long-term management’ areas ‘seed’ from *S. pedunculata* was the most important plant food source. Small Tree Finches were observed consuming plant food twice as often in the present study (66% of the foraging observations) than reported in Tebbich et al. (2004; 33% of the observations). Tebbich et al. (2004) conducted their survey from 1995 to 1998, prior to the invasion of *R. niveus*, so at that time ‘nectar’ and ‘fruit’ from this species were not available.

Our study suggests that the invasive *R. niveus* provides a new foraging substrate and food resources (‘nectar’ and ‘fruit’), especially for the Small Tree Finch. The incorporation of *R. niveus* into the species’ diet in less than two decades is another example of the flexibility of Darwin’s Finches in exploiting newly available food sources (Tebbich et al. 2004, 2008, 2010; Christensen and Kleindorfer 2008). It is known that bird-flower visitation and seed dispersal networks can lead to an accelerated spread of introduced species and if so, this could be a key factor in such species becoming invasive (Richardson et al. 2000; Bartuszevige and Gorchov 2006; Traveset et al. 2015). As *R. niveus* flowers and fruits are also part of the diet of other Darwin’s Finches and other land birds (Jewell and Buddenhagen 2006; Heleno et al. 2013), the birds can be considered as vectors for the spread of *R. niveus*.

This study has revealed novel data about foraging techniques, substrates, prey items, use of microhabitats and foraging success of arboreal Darwin’s Finches. However, prey attack rate and foraging success measured by items attacked or ingested per time does not give a direct measure of prey quality. This would require the collection and analysis of plant food sources and arthropods in the three habitat conditions. A study investigating food availability must be based on a detailed analysis of the foraging ecology of the target species (Hutto 1990; Johnson 2000). The present study indicates that the most important substrates used by both Darwin’s Finch species are dead and green leaves in the canopy, moss on tree trunks and the understory in general; hence these structures should be targeted for future arthropod sampling. Field observations suggested that moths and caterpillars were the most frequent prey items and thus they should receive specific attention. However, the analysis of stomach contents of dead chicks showed that not all prey types had been recorded during the field observations. While analysis of stomach contents confirmed caterpillars as the most abundant invertebrate prey type, ‘ants’, ‘beetles’, ‘cicadas’, ‘invertebrate eggs’ and ‘spiders’ were also found, although they were rarely recorded as prey in the field. Future studies investigating the foraging ecology of arboreal Darwin’s Finches should also consider these taxa for arthropod sampling. Another limitation of our study is that data cover only the wet, breeding season when food is more abundant (Tebbich et al. 2004). It is possible that the harsher conditions of the dry season present the real bottleneck in food abundance, which could potentially influence the survival of fledglings differentially in ‘invaded’, ‘recently controlled’ and ‘long-term management’ areas.

## Conclusion

Although microhabitat use, foraging substrate and prey choice of the Small Tree Finch and the Warbler Finch in this study differed among the habitat conditions, prey attack rate and foraging success remained similar. However, it is possible that the prey caught in each area may differ in quality (e.g. energy content and digestibility) as shown for the Grey Partridge in herbicide controlled areas in the UK (Boutin et al. 2012), which could have both short and long-term affects on adult birds and chicks. These possibilities should be investigated in future studies.

## Electronic supplementary material

Below is the link to the electronic supplementary material.

Supplementary material 1 (DOCX 14 kb)
