# Supplementary material for: The impact of invasive plant management on the foraging ecology of the Warbler Finch (Certhidea olivacea) and the Small Tree Finch (Camarhynchus parvulus) on Galápagos
Source: J Ornithol. 2017 Aug 8;159(1):129–40. doi: 10.1007/s10336-017-1481-4 (PMC6956869; doi:10.1007/s10336-017-1481-4)
Supplement: Supplementary file 1 — Supplementary material 1 (DOCX 14 kb) [file 10336_2017_1481_MOESM1_ESM.docx]

**The impact of invasive plant management on the foraging ecology of the Warbler Finch (*Certhidea olivacea*) and the Small Tree Finch (*Camarhynchus parvulus*)**

**Journal of Ornithology**

Nikolaus Filek^1^, Arno Cimadom^1^, Christian H. Schulze^2^, Heinke Jäger^3^, Sabine Tebbich^1*^

Affiliation

^1^ Department of Behavioural Biology, University of Vienna, Althanstrasse 14, 1090 Vienna, Austria

^2^ Department of Botany and Biodiversity Research, University of Vienna, Rennweg 14, 1030 Vienna, Austria

^3^ Charles Darwin Foundation, Puerto Ayora, Santa Cruz, Galapagos, Ecuador

* Correspondence to Sabine Tebbich

mail: sabine.tebbich@univie.ac.at

phone: +43-1-4277-54464

fax: +43-1-4277-854464

**Table S1** Median duration and 1^st^ and 3^rd^ quartile of foraging bouts recorded for Small Tree Finches (*Camarhynchus parvulus*) and Warbler Finches (*Certhidea olivacea*) foraging in three different habitat conditions (‘invaded,’, ‘recently controlled’ and ‘long term management’).

|  | **Duration of foraging bouts [s]** | | |  | | |
| --- | --- | --- | --- | --- | --- | --- |
|  | **Median** | **1^st^ quartile** | **3^rd^ quartile** | | **n** |  |
| **Small Tree Finch** |  |  |  | |  |  |
| Invaded | 162 | 113 | 254 | | 47 |  |
| Recently controlled | 198 | 75 | 395 | | 30 |  |
| Long term managed | 162 | 105 | 234 | | 71 |  |
|  |  |  |  | |  |  |
| **Warbler Finch** |  |  |  | |  |  |
| Invaded | 121 | 77 | 188 | | 66 |  |
| Recently controlled | 108 | 74 | 152 | | 51 |  |
| Long term managed | 132 | 101 | 174 | | 74 |  |

**Table S2** Median duration and 1^st^ and 3^rd^ quartile of foraging bouts recorded for Small Tree Finches (*Camarhynchus parvulus*) and Warbler Finches (*Certhidea olivacea*) foraging in two different microhabitats (canopy and understory).

|  | **Duration of foraging bouts [s]** | | |  | | |
| --- | --- | --- | --- | --- | --- | --- |
|  | **Median** | **1^st^ quartile** | **3^rd^ quartile** | | **n** |  |
| **Small Tree Finch** |  |  |  | |  |  |
| Canopy | 173 | 112 | 255 | | 119 |  |
| Understory | 176 | 83 | 276 | | 29 |  |
|  |  |  |  | |  |  |
| **Warbler Finch** |  |  |  | |  |  |
| Canopy | 126 | 90 | 180 | | 130 |  |
| Understory | 113 | 88 | 154 | | 61 |  |
